# Combination of Photodynamic and Bronchoscopic Intervention With Radiation Therapy in Treating Pulmonary Pleomorphic Sarcoma: A Case Report

**DOI:** 10.1111/crj.70129

**Published:** 2025-09-18

**Authors:** Ran An, Shishou Wang, Lijian Tang, Tao Feng, Chen Lin, Chongqing Lv

**Affiliations:** ^1^ Department of Respiratory and Critical Care Medicine Shengli Oilfield Central Hospital Dongying City Shandong Province China; ^2^ Hepatobiliary Surgery Shengli Oilfield Central Hospital Dongying City Shandong Province China

**Keywords:** photodynamic therapy, primary pulmonary synovial sarcoma, radiation

## Abstract

Primary pulmonary synovial sarcoma (PPSS) is a rare and challenging malignancy in terms of diagnosis and treatment. We narrated a case of PPSS treated innovatively by integrating photodynamic therapy (PDT) and localized radiation, overcoming diagnostic and treatment challenges associated with PPSS. The combination significantly improved clinical symptoms and patient survival. The case sheds light on a promising therapeutic strategy, augmenting the treatment discourse for PPSS and potentially guiding future clinical practices in managing this rare malignancy.

## Graphical abstract

We discuss a case where an innovative combination of photodynamic therapy (PDT) and local radiation therapy to address the diagnostic and therapeutic challenges associated with PPSS resulted in significant improvements in clinical symptoms and patient survival, demonstrating the success of this treatment.

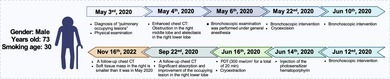

## 1 Introduction

Primary pulmonary synovial sarcoma (PPSS) is a rare soft tissue tumor, accounting for 8% of all soft tissue sarcomas [1]. It originates not from the synovial tissue but from mesenchymal tissue [2, 3] and is most commonly found in the extremities, especially near large joints, leading to its misidentification as being synovial in origin. Reports have shown that synovial sarcomas also occur in the lungs, mediastinum, abdomen, head and neck, and heart [4, 5]. Metastatic synovial sarcomas from the extremities are most commonly found in the lung parenchyma and pleura. Among all primary pulmonary malignancies, lung sarcomas account for 0.5%, with malignant fibrous histiocytomas and synovial sarcomas being the most common [6]. Despite its high sensitivity, molecular testing is unnecessary if synovial sarcoma is confirmed or likely based on clinical symptoms, histology, and immunohistochemistry. Research indicates that two‐thirds of PPSS is central in type, associated with airway obstruction symptoms such as cough, dyspnea, fever, and hemoptysis [7]. Peripheral tumors are less common, typically asymptomatic, but can invade nearby tissues (pleura, chest wall, and mediastinum) or cause distant metastases, for example, to the adrenal glands, brain, and spinal cord [7]. Computerized tomography (CT) of PPSS includes a well‐defined large mass in the lung with soft tissue density. The mass may show mild to moderate enhancement, with or without mediastinal lymph node enlargement [8]. Efficiently diagnosing and treating PPSS have become an urgent issue that needs addressing.

In recent years, photodynamic therapy (PDT) has emerged as a novel treatment modality, showing significant promise in tumor management due to its minimally invasive nature, fewer side effects, and ability to precisely eradicate tumor cells [9, 10]. PDT employs a photosensitizer, which, when activated by light of a specific wavelength, generates reactive oxygen species mediating cytotoxicity [11, 12, 13]. PDT kills cells directly through apoptosis or necrosis, disrupts tumor vasculature, and may induce inflammatory responses, thereby triggering host anti‐tumor immune reactions [14]. The common light source for PDT is laser, which can be guided by optical fibers and introduced into the lungs through bronchoscopy for lung cancer treatment [15]. Sodium porfimer is the most commonly used photosensitizer for treating pulmonary malignancies [16, 17] and can be employed to alleviate airway obstruction symptoms in patients with non‐small‐cell lung cancer (NSCLC) when standard treatments fail. PDT offers several advantages for treating early or advanced NSCLC compared with other modalities: It can be readministered for diseases that are not fully relieved or have recurred, and mutations conferring resistance to radiation or chemotherapy do not limit the efficacy of PDT.

**FIGURE 1:**
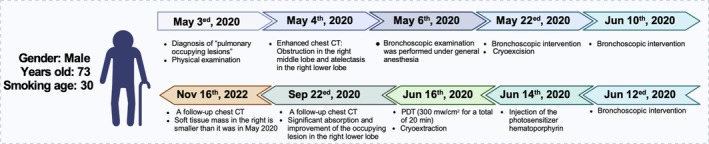
Treatment chart.

This paper discusses a case of an elderly male with PPSS (Figure 1). Through bronchoscopic biopsy, we were able to pathologically confirm the diagnosis of PPSS. Faced with the patient's refusal of surgery and chemotherapy, and considering the malignant nature and rapid progression of the disease, we adopted a multimodal treatment approach of bronchoscopic interventions combined with photodynamics and local radiation. This effectively improved the patient's clinical symptoms and significantly extended the survival of the PPSS patient, unveiling a novel and promising combined treatment scheme for PPSS patients. Furthermore, we delved into the application of PDT in the realm of pulmonary tumor treatment, providing valuable references and insights for future clinical treatments.

## 2 Case presentations

The patient, a 73‐year‐old male with a smoking history of 30 years, sought medical attention on 3 May 2020, due to “shortness of breath and cough with phlegm following exertion for 1 month.” Since April 2020, without any apparent precipitating factors, the patient had been experiencing breathlessness following exertion, accompanied by cough and a small amount of white sticky phlegm. He was given “anti‐inflammatory” treatment (specifics unknown) outside the hospital, which proved ineffective.

A chest CT on 3 May 2020 revealed a mass in the right lower lobe and atelectasis, enlarged lymph nodes at the right hilum, raising the suspicion of lung cancer. He was admitted to our department with a diagnosis of “pulmonary occupying lesions.” He had a 6‐year history of “carotid artery stenosis” and had been on a regular medication regimen of “aspirin and Naoxintong capsule.” Neither personal nor family medical history presented any notable findings.

Upon admission, the physical examination showed: body temperature 36.8°C, pulse 90 beats/min, and blood pressure 128/85 mmHg (1 mgHg = 0.133 kPa). The patient was alert, with a normal mental status; no superficial lymphadenopathy was noted; coarse breath sounds were heard in both lungs without fine or coarse crackles; cardiac and abdominal examinations were unremarkable. Complete blood count, coagulation profile + D‐dimer, and male tumor markers were within normal limits.

**FIGURE 2:**
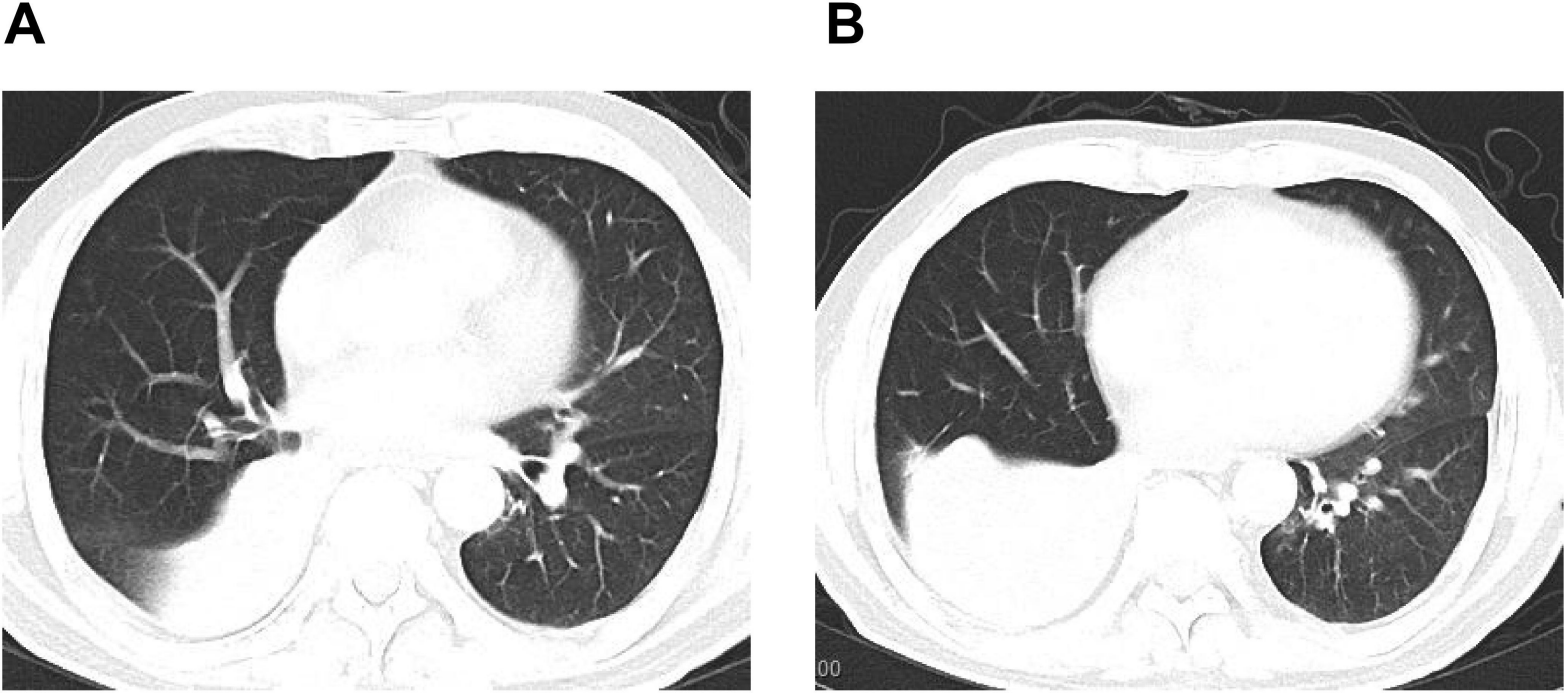
Enhanced chest CT on 4 May 2020. (A) The showed bronchus occlusion in the lower lobe of the right lung, with soft tissue nodules in it, and consolidation of the distal lung tissue with flacke‐like soft tissue masses (B).

An enhanced chest CT on 4 May 2020, as shown in Figure 2, indicated obstruction in the right middle lobe and atelectasis in the right lower lobe. To further clarify the diagnosis, bronchoscopic examination was performed under general anesthesia on 6 May 2020. Intraoperatively, congestion and edema of the bronchial mucosa were observed at the junction of the right upper lobe and right middle lobe bronchi. The lumen at the starting point of the right intermediate bronchus was obstructed by a neoplasm with a necrotic surface. Utilizing a combination of snare, cryotherapy, and APC (argon plasma coagulation), tumor tissues were cleared, exposing the bronchus of the right middle lobe and the dorsal segment bronchus of the right lower lobe, but the right basal segment bronchus remained obscured.

**FIGURE 3:**
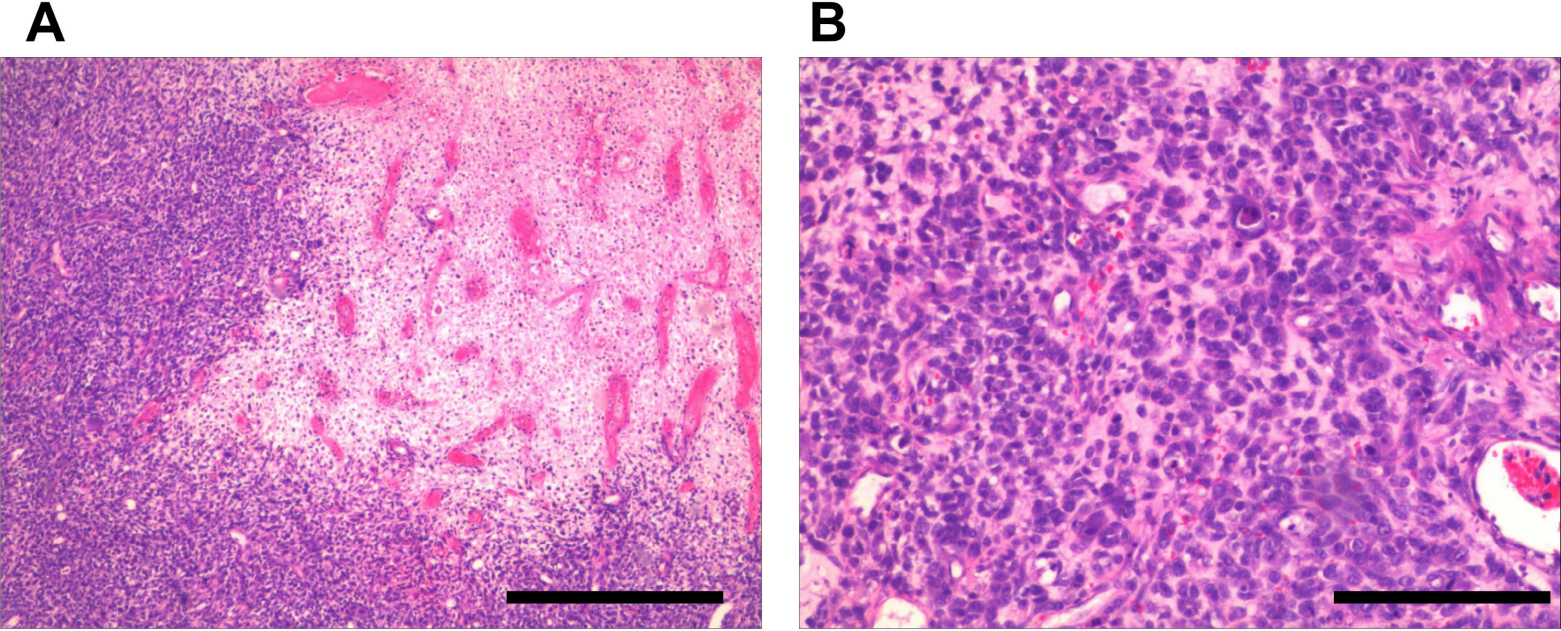
Pathological H&E staining results of bronchoscopic biopsy of the middle segment of right lung. (A) Four‐fold image. (B) Ten‐fold image. Scale bar: 1.00 mm.

Bronchoscopic biopsy of the right middle lobe bronchus revealed: Under light microscopy, epithelial and spindle cell components were visible. The epithelial area contained slit‐like glandular spaces, with cells appearing cuboidal, cytoplasm showing eosinophilia, and nuclei round or oval with granular chromatin, visible nucleoli, and frequent mitotic figures. Necrosis was seen surrounding the tumor tissue. In the spindle cell area, cells appeared oval, intertwined densely in bundles, accompanied by mucoid regions (Figure 3A,B).

The bronchoscopic pathology report indicated that the biopsy from the right middle lobe bronchus showed a malignant tumor. Coupled with histological morphology and immunohistochemical results, it confirmed the diagnosis of poorly differentiated synovial sarcoma with local patchy tumor necrosis, thereby confirming the diagnosis of PPSS.

**FIGURE 4:**
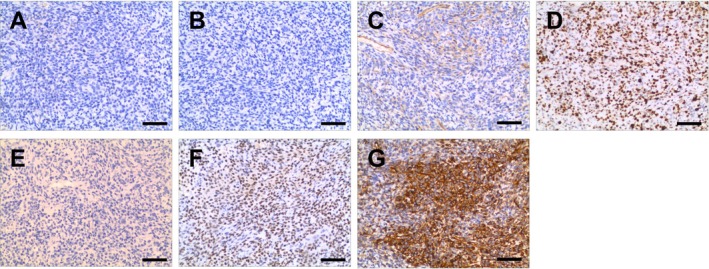
Results of immunohistochemistry. Immunohistochemistry analysis of Bcl‐2 (A), CD99 (B), CK (C), ki‐67 (D), s100 (E), TLE1 (F), and Vimentin (G) expression in tumor tissues. Scale bar: 50 μm.

Immunohistochemical results were as follows: Broad‐spectrum CK(−), CAM5.2 (−), EMA(−), Vimentin(3+), CD117(−), CD56(−), CgA(−), CK20(−), CK5/6(−), CK7(−), β‐catenin(+), NapsinA(−), P40(−), PD‐1 (−), PD‐L1(−), Syn(−), TTF(−), CD99(−), Desmin(−), S‐100(−), Bcl‐2 (−), CD34(−), TLE‐1(3+), HMB45(−), P63(−), TLE‐1(3+), HBME‐1(−), CK7(−), CK19(−), STAT‐6(−), Ki67(+, 65–70%) (Figure 4).

Enhanced PET‐CT completed on 19 May 2020: Mildly increased glucose metabolism associated with lymph nodes in the mediastinal zones 4R, 5, 7, and 10 L, with a maximum short diameter of approximately 9.0 mm and a maximum SUV value of about 2.9. The right lung's intermediate bronchus is obstructed, with unclear visualization of the dorsal and various basal segment bronchi, measuring approximately 77.3 mm × 42.7 mm × 73.5 mm; the maximum SUV values range from 7.4 to 8.5. Within the lesion, irregular areas of decreased glucose metabolism and low density are observed, with a crescent‐like dense shadow in the posterior part, and no increased glucose metabolism is seen. The trachea and left lung's lobar bronchi are patent, with no obvious abnormal density or increased glucose metabolism in the left lung. A fluid low density is seen in the right thoracic cavity. The diagnosis of synovial sarcoma in the patient's right lung is confirmed. Although no distant metastasis is observed, the tumor diameter is > 5 cm, which poses significant surgical challenges. After discussing with the patient's family, they decided to decline surgery and chemotherapy treatment. It is worth noting that the patient chose to refuse these traditional treatments due to concerns about severe side effects such as nausea, vomiting, fever, fatigue, and body aches. Given the high malignancy and rapid progression of this disease, we decided to adopt a combination of bronchoscopic interventions, PDT, and local radiation.

**FIGURE 5:**
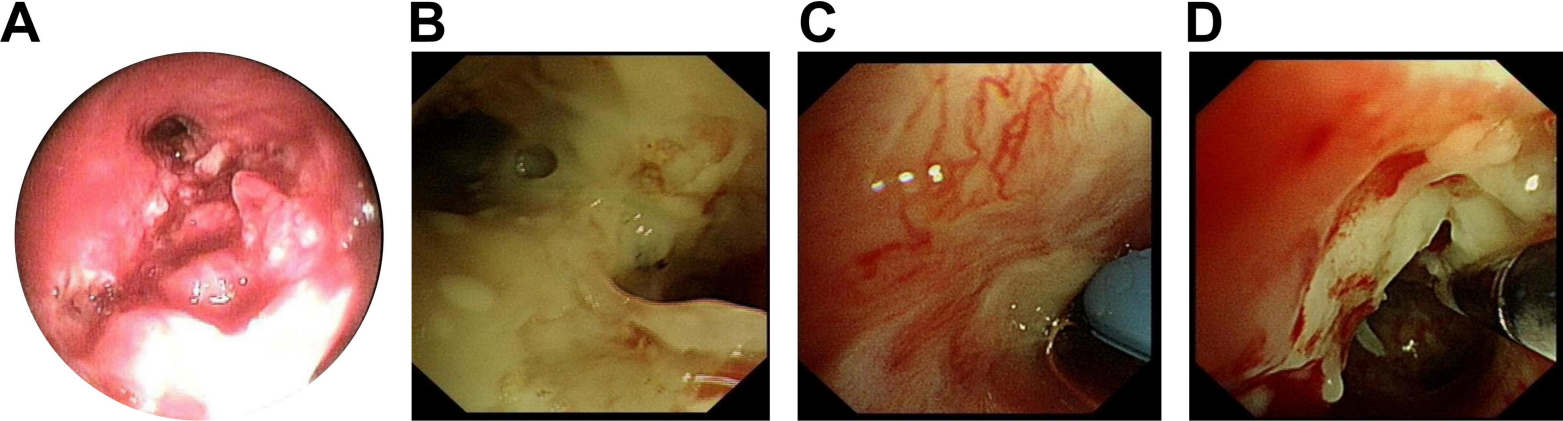
22 May 2020 bronchoscopic examination results. (A) Tumor‐like tissue was found in the right middle bronchus. (B) Necrotic material attachment was seen on the surface of the right intermediate bronchus. (C and D) Removal of necrotic tumor tissue using foreign body forceps under bronchoscopy.

On 22 May 2020, a bronchoscopic intervention was performed. Under bronchoscopy, the left lung lobes were patent, the right upper lobe was clear, while neoplastic and necrotic tissues were observed in the right middle lobe, obstructing the lumen (Figure 5A,B). After cryoexcision and the use of foreign body forceps to remove the tumor tissue (Figure 5C,D), the bronchial lumen of the right middle lobe was observed to be narrowed, and the dorsal segment was obstructed, with the bronchoscope unable to pass through. The neoplasm originated from the basal segment of the right lower lobe. Following the cryoexcision, hemostasis was achieved using APC, and the procedure was then concluded. For further clearance of tumor tissues, bronchoscopic interventions were carried out again on 10 June and 12 June 2020. Intraoperatively, tumor‐like tissues obstructed the right intermediate bronchus, causing lumen narrowing. Using cryotherapy, biopsy forceps, and foreign body forceps, tumor tissues were cleared, and the right intermediate bronchus was unobstructed, though the right lower lobe bronchus remained blocked.

Considering the patient's diagnosis of pulmonary synovial sarcoma and the poor efficacy of radiochemotherapy, local treatment was prioritized. PDT was planned, and on 14 June 2020, the patient was administered an intravenous injection of the photosensitizer hematoporphyrin at 175 mg (2 mg/kg) and was advised to strictly avoid light exposure.

**FIGURE 6:**
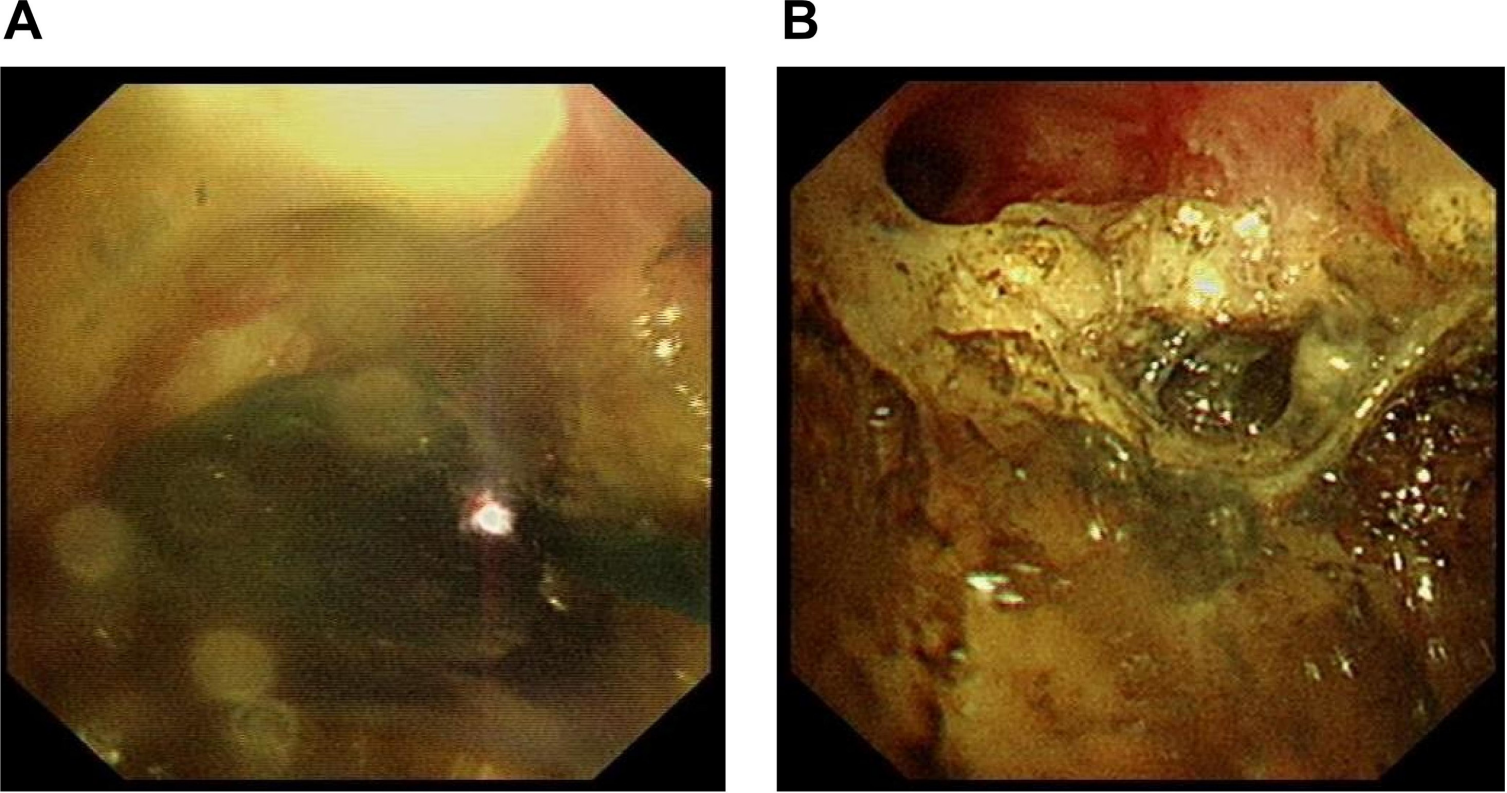
PDT. (A) Photodynamic treatment of right intermediate bronchus. (B) Right intermediate bronchus after PDT.

**FIGURE 7:**
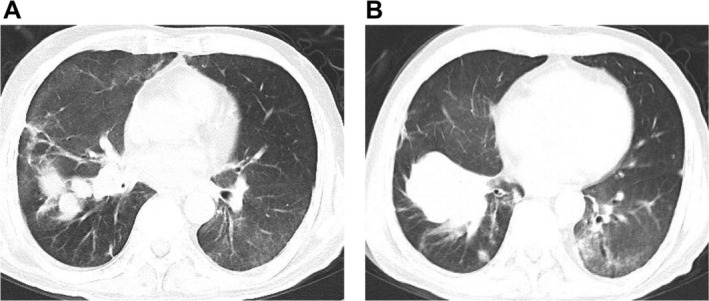
Chest CT on 20 September 2020. The soft tissue mass shadow within the bronchus of the right lower lobe and the shadow of solid changes in the distal lung tissue have both decreased in size compared with previous assessments.

**FIGURE 8:**
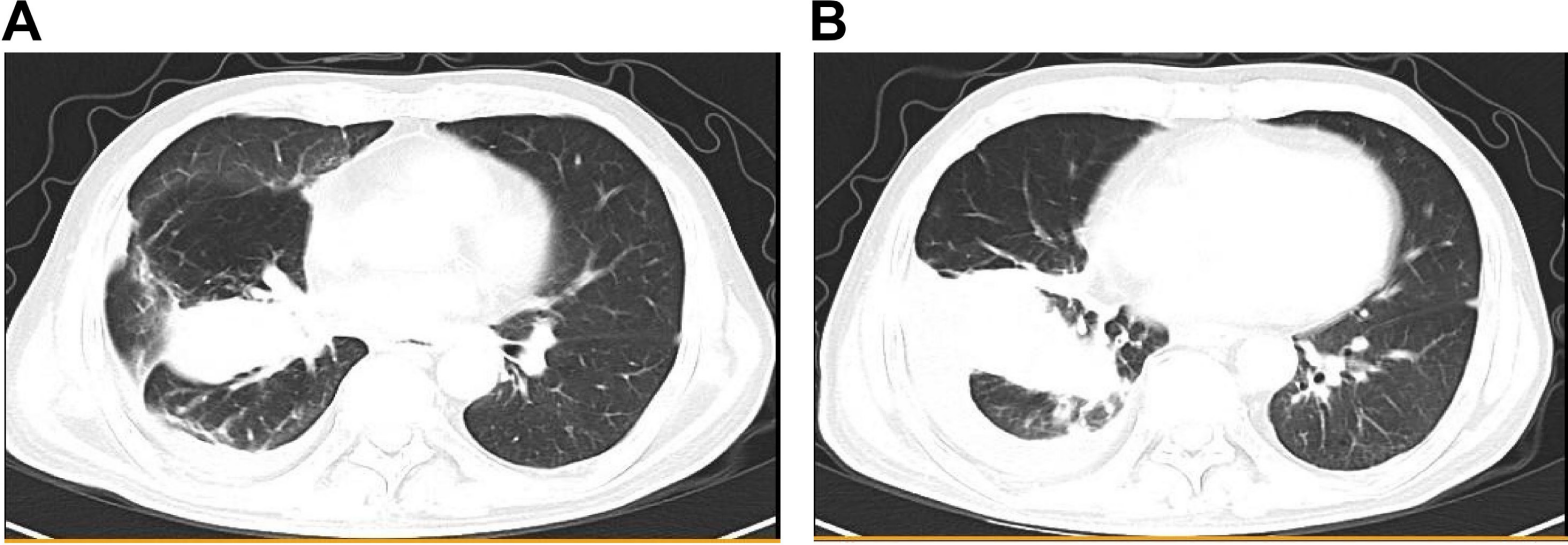
Chest CT on 16 October 2022. The space‐occupying lesion in the right lower lobe has decreased in size compared with 4 May 2020, while the range of obstructive pneumonia in the right lower lobe has increased compared with before. Remaining bilateral lung inflammation has improved since the previous assessment, and the pleural effusion on the right side has decreased in extent.

On 16 June 2020, under general anesthesia, cryoextraction was carried out in the right middle lobe and necrotic material within the right middle lobe was removed, followed by PDT. A 630‐nm laser fiber was placed in the right middle lobe bronchus, delivering an energy of 300 mw/cm^2^ for a total of 20 min; similar irradiation was performed in the right intermediate bronchus. Bronchoscopic observation revealed rapid tumor tissue growth in the short term; after repeated cryotherapy and electroresection, the right intermediate bronchus was more patent than before treatment, with lumen expansion. However, the right lower lobe bronchus remained obstructed by tumor tissues, and local radiation was suggested. The patient underwent intermittent radiation combined with bronchoscopic interventions and PDT (Figure 6). A follow‐up chest CT on 22 September 2020 (Figure 7) showed significant absorption and improvement of the occupying lesion in the right lower lobe compared with before. Preoperative lung function: FEV1/FVC 60.45%, FEV1 is 70.66% of the predicted value. Postoperative lung function: FEV1/FVC 75.35%, FEV1 is 80.57% of the predicted value. Two years after the above treatments, a follow‐up chest CT on 16 October 2022 (Figure 8) showed the soft tissue mass in the right lower lobe measuring approximately 6.6 cm × 4.6 cm, a decrease from May 2020. Currently, the patient is followed up by phone, and their general condition is acceptable, with no shortness of breath.

Two years post‐treatment, the patient's general condition is acceptable, with no notable breathlessness. Follow‐up chest CT showed relatively stable lesions, and the patient is currently under outpatient follow‐up. Through this combined treatment approach, we successfully improved the patient's clinical symptoms, extended the survival of the PPSS patient, and provided a new combined treatment modality for PPSS patients. This new treatment combination reduced the invasiveness and side effects of treatment, offering patients a broader range of treatment options and also providing a valuable reference for the future clinical management of PPSS.

## 3 Discussion

PPSS is a highly aggressive pulmonary tumor [18]. It was first described by Zeren et al. [19] in 1995, manifesting as chest pain, cough, shortness of breath, hemoptysis, or ipsilateral pleural effusion, with sometimes a massive mass visible within the thoracic cavity. Unlike other pulmonary sarcomas, it mainly comprises two morphologically distinct cells: epithelial cells or fibroblast‐like spindle cells [20]. Histopathologically, it is categorized into four types: biphasic, monophasic, monophasic epithelial, and poorly differentiated [21]. Monophasic synovial sarcoma is the most common type and is considered the classical type [22]. The epithelial cells are characterized by large, round, or oval vesicular nuclei, abundant pale cytoplasm, and clear cell boundaries [23], while spindle cells have uniform morphology, oval or dark‐stained nuclei, scarce cytoplasm, and indistinct borders. Mitotic figures can be seen in both epithelial and spindle cells. Monophasic synovial sarcoma is characterized by a predominance of spindle cells with immunopositivity for CK and EMA, and only a small number of epithelial cells. Monophasic epithelial type presents with a predominance of epithelial cells forming glandular structures, making it challenging to distinguish from lung metastatic or primary carcinoma. The diagnosis of poorly differentiated synovial sarcoma is very challenging due to its microscopic appearance of tightly arranged oval or spindle‐shaped small cells with features intermediate between epithelial and spindle cells, scant differentiation, resembling small cell lung carcinoma [21]. It may also be confused with other malignant spindle cell tumors such as fibrosarcoma, angioepithelioma, leiomyosarcoma, and spindle cell carcinoma or carcinosarcoma [22, 24, 25]. Immunohistochemistry plays a crucial role in the diagnosis of synovial sarcoma, especially in monophasic cases. Most synovial sarcomas show an immunoreaction for cytokeratin and epithelial membrane antigen (EMA) [26] The positive rates for S‐100 protein, CD99, and Bcl2 are 30%, 60%–70%, and 75%–100%, respectively [27]. Cytogenetics also plays a significant role in synovial sarcoma as both monophasic and biphasic types are characterized by reciprocal chromosomal translocations (x;18)(p11.2;q11.2) [28], translocating the SYT of chromosome 18 to either of the two homologous genes SSX1 or SSX2 on Xp11 [28]. Differential diagnosis through bronchoscopy, including lung carcinoma, mesothelioma, lymphoma, Wegener's granuloma, rheumatoid nodules, histiocytosis, and cryptococcosis, will aid in confirming PPSS. The prognosis of PPSS is very poor due to its high aggressiveness, with a 2‐year survival rate of less than 20% [29]. Adverse prognostic factors include tumor size > 5 cm, male gender, age > 20 years, extensive tumor necrosis, numerous mitotic figures (> 10/10 high‐power fields), neurovascular invasion, and SYT‐SSX1 translocation [30]. Due to its rarity and lack of related data, there are no optimal treatment guidelines at present. Surgical resection combined with adjuvant radiotherapy is currently the standard treatment modality for surgically resectable synovial sarcoma [31]. If the tumor is large (maximum diameter > 5 cm), deeply located, or invades surrounding tissues, nerves, or major blood vessels, making resection difficult, preoperative radiotherapy can be administered to reduce the size of the tumor. If the tumor cannot be completely resected during surgery, postoperative adjuvant radiotherapy is necessary to eliminate any residual tumor cells. Synovial sarcoma is relatively sensitive to chemotherapy, which is an important adjunctive treatment approach. This can be categorized into neoadjuvant chemotherapy, adjuvant chemotherapy, and palliative chemotherapy. Neoadjuvant chemotherapy may be conducted concurrently with preoperative adjuvant radiotherapy. Typically, doxorubicin plus ifosfamide or the MAID regimen (mesna, adriamycin, ifosfamide, and dacarbazine) are recommended. Postoperative adjuvant chemotherapy is strongly advised in cases where the tumor is deeply situated, has a maximum diameter > 5 cm, could not be completely resected, or where local recurrence necessitates a second resection. For adult synovial sarcoma, the adriamycin + ifosfamide regimen is generally recommended, with six cycles of chemotherapy advised. If a patient has failed multiple lines of chemotherapy and it is difficult to benefit further from chemotherapy, and if the ECOG‐PS score is > 1, further chemotherapy is not recommended. Other advanced treatment options include targeted therapy and immunotherapy, although some are still under clinical trials [32]. In this case, the patient's tumor diameter was > 5 cm and involved multiple lung lobes. Surgical resection was not effective, and the family refused surgery and chemotherapy. Therefore, we opted for bronchoscopic interventional treatment combined with PDT and localized radiotherapy to reduce tumor mass and effectively extend the patient's survival period.

PDT for treating malignant lung tumors depends on the tumor's location and size [33]. The usage of PDT has increased in the treatment of bronchogenic carcinoma following its preliminary approval by the US Food and Drug Administration for treating patients with NSCLC [34, 35]. Clinical indications for PDT in treating pulmonary NSCLC include treatment of early central endobronchial tumors; treatment of centrally recurrent tumors post‐surgery or radiation; treatment of radiographically occult central tumors and early peripheral pulmonary tumors; alleviating endobronchial obstruction and tumor‐induced airway narrowing to improve respiratory function, addressing acute hemoptysis and obstructive pneumonia, and as neoadjuvant therapy to reduce the surgical resection scope; treatment of locally advanced tumors and pleural metastatic diseases. Numerous studies have demonstrated that PDT can successfully alleviate symptomatic locally advanced NSCLC patients [36, 37, 38]. Researchers from the Northwestern University Medical School treated 10 patients with inoperable late‐stage endobronchial obstructive NSCLC with PDT between 1985 and 1989, reducing airway obstruction from 86% ± 12% to 57% ± 3%, with all patients showing symptom alleviation [39]. A prospective UK study assessed the alleviation effects of PDT on 100 patients with IIIa‐IV stage inoperable bronchial obstruction, 90% of whom had NSCLC and 82% had prior chemotherapy or radiation treatment. Post‐PDT, average intraluminal obstruction decreased from 85% to 17.5%, with similar improvements in Forced Vital Capacity (FVC) and Forced Expiratory Volume in 1 s (FEV1). The cohort had an average survival of 9 months, with a 2‐year overall survival rate of 19% [40]. Russian researchers also showed that despite two patients relapsing within 3 months post‐PDT, PDT reduced bronchial obstruction in 75% (9/12) of IIIb‐IV stage patients and achieved a 100% complete remission rate (8/8) in early‐stage cancer [41]. PDT is highly effective as a standalone treatment for lung cancers that are visible through bronchoscopy, have a size of ≤ 1 cm, and show no invasion beyond the cartilage. In cases of advanced NSCLC, this therapy can alleviate obstructions from endobronchial lesions, serve as an element in comprehensive multimodality treatment, or enhance the feasibility of surgery and potentially lessen the surgical scope needed [42].

PDT can also improve surgical outcomes, enabling locally advanced NSCLC patients the opportunity for surgical resection. Russian researchers treated 191 patients with bronchoscopic surgery, including 153 with advanced NSCLC causing respiratory obstruction. Incorporating PDT in the surgery could possibly improve patient prognosis compared with surgery alone [43]. Although PDT has limitations including photosensitivity reactions post‐treatment [44, 45] and bronchogenic tumors invading the pulmonary artery being ineligible for treatment, PDT has been successfully used to reduce the surgical resection scope, enabling initially planned pneumonectomy patients to undergo lobectomy instead, and providing surgical opportunities for otherwise inoperable patients [46]. At Tokyo Medical University Hospital, 85% of 26 NSCLC patients who received preoperative PDT to shrink tumor resection scope or secure surgical chances achieved their surgical goals. Four out of five initially inoperable patients underwent surgery, and 18 out of 21 initially pneumonectomy‐only patients were able to have lobectomy [46]. PDT can also be part of the treatment for NSCLC with pleural metastasis. A phase II trial conducted by the University of Pennsylvania included 22 patients with pleural metastasis and clinical T4 NSCLC. Patients underwent complete (*n* = 17) or partial tumor resection (*n* = 5), followed by unilateral pleural PDT (*n* = 20) or PDT alone (n = 2). The cohort had a 6‐month local control rate of 73.3% and a median overall survival of 21.7 months, compared with a historical control survival of 6–9 months in similar patients [47]. Prognosis is poor for advanced and metastatic NSCLC patients, who often require palliative symptomatic treatment. Although these patients have harder‐to‐achieve satisfactory treatment outcomes compared with early‐stage lung cancer patients, PDT can effectively alleviate their symptoms [48, 49]. PDT can reduce endobronchial obstruction and tumor‐induced airway narrowing, thus improving patients' dyspnea and lung function [50]. PDT can also address acute hemoptysis and obstructive pneumonia, improving patients' physical status [51]. Additionally, when used as neoadjuvant therapy, PDT can enhance operability or reduce the required surgical scope, while also potentially extending the survival of NSCLC patients with pleural metastasis [52].

In this case, PDT emerges as a relatively new and promising anti‐lung tumor treatment method, although it has not been fully utilized and popularized to date. Immunohistochemistry plays a critical role in diagnosing PPSS, especially in monophasic cases. The presence of specific chromosomal translocations (x;18) is a hallmark of this sarcoma. Currently, PDT for the treatment of NSCLC demonstrates good tolerance and minimal severe toxicity, becoming a potential treatment option. In the treatment of early‐stage NSCLC, for instance, occult cancers or primary bronchogenic carcinomas displayed on X‐rays, PDT may become the sole treatment option. For late‐stage lung cancer patients, PDT effectively alleviates symptoms caused by airway obstruction, improving patients' respiratory function and quality of life. Research suggests that PDT combined with radiation therapy could be a viable treatment option for obstructive NSCLC patients. Although the patient number is extremely limited (*n* = 11), a randomized trial was completed in Vancouver to ascertain if PDT combined with 30 Gy of external radiation therapy (split over 10 sessions, 2 weeks) could improve the prognosis of inoperable central NSCLC patients. All patients saw symptom improvement 4 weeks post‐treatment. In later follow‐ups, based on post‐treatment pulmonary function tests, respiration‐perfusion lung scans, CT scans, bronchoscopic examinations, and quality of life scores, it was found that adding PDT before radiation therapy significantly improved patient symptoms and controlled tumor progression [53]. Additionally, 41 locally advanced NSCLC patients (including 78% with Stage III disease) at The Ohio State University Medical Center underwent induction PDT and chemotherapy and/or radiation. PDT‐based induction enabled 57% of initially deemed unresectable patients to undergo curative surgery, 27% of initially deemed pneumonectomy‐needed patients to undergo lobectomy, 64% of patients achieved pathological downstaging, and 18% underwent surgery with pathology showing complete remission. Overall, 46% of patients were still alive within 3 years post‐treatment, with those undergoing lobectomy having the longest average survival (35.9 months), and inoperable patients having the shortest average survival (14.7 months) [48]. Therefore, administering PDT before radiation shows good local control over the tumor. Simultaneously, PDT can serve as an induction therapy before chemotherapy, increasing the tumor's resectability and reducing the required surgical scope.

In this instance, the patient initially presented with breathlessness post‐activity, followed by chest imaging showing a soft tissue mass in the right lower lobe bronchus with distal lung tissue consolidation. Ultimately, PPSS was pathologically confirmed through bronchoscopic biopsy. The patient refused surgery and chemotherapy treatment. We employed bronchoscopic cryotherapy, biopsy, and snare cutting methods to clear the tumor tissue, reopening the right intermediate bronchus, but the right lower lobe bronchus remained obstructed. Therefore, we considered applying photodynamic palliation to alleviate the patient's symptoms, combined with local radiation to control tumor growth. Through various combined treatments, good control over PPSS was achieved.

For lung tumor patients, the approach of combining PDT with bronchoscopic intervention or local radiation for treating PPSS is a relatively new and promising anti‐tumor treatment method, yet to be fully utilized. Our results show that PDT for treating NSCLC generally has good tolerance with rarely severe toxicity. For early‐stage NSCLC such as occult cancers or primary bronchogenic carcinomas on X‐rays, PDT can serve as a standalone therapy, while for late‐stage NSCLC, PDT can alleviate symptoms of airway obstruction, and it can be combined with bronchoscopic intervention and/or local radiation to control lung tumor growth, extending the survival period for advanced tumor patients. PDT can also serve as neoadjuvant therapy, increasing surgical operability or reducing the required surgical scope, offering a diversified choice for clinical treatment.

## 4 Conclusions

Our findings indicate that the application of PDT extends beyond solitary treatment. It can be amalgamated with bronchoscopic intervention and/or local radiotherapy to jointly control the growth of lung tumors, thereby prolonging the survival period of late‐stage tumor patients. PDT can serve as a neoadjuvant treatment measure, enhancing the operability of tumors or reducing surgical trauma, offering a diverse array of choices for clinical treatment. This allows patients to receive more personalized and comprehensive treatment plans. In the specific case discussed in this text, the application of PDT significantly alleviated the clinical symptoms of the patient. Combined with local radiotherapy, the multimodal treatment regimen effectively controlled the progression of PPSS, showcasing the significant value of PDT in combined treatment plans. This combined treatment approach not only provides new treatment options for PPSS patients but also offers a broader range of treatment choices and references for clinical practitioners when faced with complex and rare cases.

PDT is analogous to physical therapy, with the following potential side effects: (1) Photoallergic reactions: Some patients may exhibit localized erythema and skin itching after premature exposure to light, predisposing them to skin allergies. (2) During the treatment of respiratory system tumors, patients may experience coughing and expectoration. Failure to timely clear necrotic debris from the airways can lead to respiratory distress; in rare cases, obstruction of the duct by necrotic material may cause pulmonary infections. (3) There is an increased risk of fistula formation following tumor necrosis and detachment. (4) Tumor necrosis and the formation of local mucosal scars can lead to luminal narrowing. After undergoing PDT, patients are required to avoid light exposure for approximately 1 month and must undergo timely bronchoscopic cleaning to ensure airway patency [54].

In summary, this case report underscores the potential and value of PDT in the treatment of lung tumors, whether as a standalone treatment modality or in combination with other treatment methods. This includes its application in rare and challenging cases such as PPSS, showcasing the important position of PDT in modern comprehensive treatment of lung tumors. By delving deeper and exploring more possibilities with PDT, it helps in providing safer, more effective, and diversified treatment options for lung tumor patients. This also provides beneficial references and insights for the development of comprehensive treatment approaches for lung tumors in the future.

## Author Contributions

Ran An, Shishou Wang, Chen Lin, and Chongqing Lv: manuscript writing, investigation, methodology, and validation; Tao Feng and Chen Lin: data collection, data curation, and analysis; Chen Lin and Chongqing Lv: data curation and analysis and draft editing; Ran An, Shishou Wang, Chen Lin, and Chongqing Lv: study conceptualization, supervision, reviewing, manuscript writing, revising, and editing. Ran An and Shishou Wang made the same contribution to this work and should share the first authorship. All authors have read and approved the final version of the manuscript.

## Ethics Statement

The studies involving human participants were reviewed and approved by Shengli Oilfield Central Hospital. The patients/participants provided their written informed consent to participate in this study. Written informed consent was obtained from the individual(s) for the publication of any potentially identifiable images or data included in this article. The authors are accountable for all aspects of the work in ensuring that questions related to the accuracy or integrity of any part of the work are appropriately investigated and resolved. The study was conducted in accordance with the Declaration of Helsinki (as revised in 2013). The study was approved by the Institutional Review Board of Shengli Oilfield Central Hospital. All the study subjects provided informed consent.

## Conflicts of Interest

The authors declare no conflicts of interest.

## Data Availability

Not applicable.

## References

[1] N. Fujimoto , T. Kubo , M. Hisaoka , et al., “Demographics, Management and Treatment Outcomes of Benign and Malignant Retroperitoneal Tumors in Japan,” International Journal of Urology 25 (2018): 61–67. 28994196 10.1111/iju.13469

[2] M. Mirzoyan , A. Muslimani , S. Setrakian , M. Swedeh , and H. A. Daw , “Primary Pleuropulmonary Synovial Sarcoma,” Clinical Lung Cancer 9 (2008): 257–261. 18824448 10.3816/CLC.2008.n.040

[3] S. L. Spunt , L. Million , Y.‐Y. Chi , et al., “A Risk‐Based Treatment Strategy for Non‐Rhabdomyosarcoma Soft‐Tissue Sarcomas in Patients Younger Than 30 Years (ARST0332): A Children's Oncology Group Prospective Study,” Lancet Oncology 21 (2020): 145–161. 31786124 10.1016/S1470-2045(19)30672-2 PMC6946838

[4] S. Kumar , K. Goyal , R. Bhatt , S. Bansal , and M. Mishra , “Primary Pleural Synovial Sarcoma: A Rare Cause of Hemorrhagic Pleural Effusion,” Advances in Respiratory Medicine 89 (2021): 60–62. 33471348 10.5603/ARM.a2020.0147

[5] J.‐Y. Blay , M. von Mehren , R. L. Jones , et al., “Synovial Sarcoma: Characteristics, Challenges, and Evolving Therapeutic Strategies,” ESMO Open 8 (2023): 101618. 37625194 10.1016/j.esmoop.2023.101618 PMC10470271

[6] U. B. Shah , S. Joshi , S. V. Ghorpade , S. N. Gaikwad , and R. M. Sundrani , “Primary Pleuro‐Pulmonary Synovial Sarcoma,” Indian Journal of Chest Diseases & Allied Sciences 52 (2010): 169–172. 20949738

[7] B. Etienne‐Mastroianni , L. Falchero , L. Chalabreysse , et al., “Primary Sarcomas of the Lung: A Clinicopathologic Study of 12 Cases,” Lung Cancer 38 (2002): 283–289. 12445750 10.1016/s0169-5002(02)00303-3

[8] D. J. Boulter , M. L. Rosado‐de‐Christenson , R. Stevens , and S. Suster , “Primary Synovial Sarcoma of the Lung,” Radiology Case Reports 2 (2007): 82. 27303489 10.2484/rcr.v2i4.82 PMC4895772

[9] S.‐A. Son , J.‐H. Kim , and J.‐K. Park , “Clinical Applications of a Quantitative Light‐Induced Fluorescent (QLF) Device in the Detection and Management of Cracked Teeth: A Case Report,” Photodiagnosis and Photodynamic Therapy 43 (2023): 103735. 37544373 10.1016/j.pdpdt.2023.103735

[10] G. Xu , C. Li , C. Chi , et al., “A Supramolecular Photosensitizer Derived From an Arene‐Ru (II) Complex Self‐Assembly for NIR Activated Photodynamic and Photothermal Therapy,” Nature Communications 13 (2022): 3064. 10.1038/s41467-022-30721-w PMC9163081 35654794

[11] S. Moghassemi , A. Dadashzadeh , R. B. Azevedo , and C. A. Amorim , “Nanoemulsion Applications in Photodynamic Therapy,” Journal of Controlled Release 351 (2022): 164–173. 36165834 10.1016/j.jconrel.2022.09.035

[12] H. Abrahamse and M. R. Hamblin , “New Photosensitizers for Photodynamic Therapy,” Biochemical Journal 473 (2016): 347–364. 26862179 10.1042/BJ20150942 PMC4811612

[13] M. Lan , S. Zhao , W. Liu , C.‐S. Lee , W. Zhang , and P. Wang , “Photosensitizers for Photodynamic Therapy,” Advanced Healthcare Materials 8 (2019): e1900132. 31067008 10.1002/adhm.201900132

[14] P. Agostinis , K. Berg , K. A. Cengel , et al., “Photodynamic Therapy of Cancer: An Update,” CA: A Cancer Journal for Clinicians 61 (2011): 250–281. 21617154 10.3322/caac.20114 PMC3209659

[15] M. B. Vrouenraets , G. W. M. Visser , G. B. Snow , and G. A. M. S. van Dongen , “Basic Principles, Applications in Oncology and Improved Selectivity of Photodynamic Therapy,” Anticancer Research 23 (2003): 505–522. 12680139

[16] C. Nwogu , A. Kloc , K. Attwood , W. Bshara , F. Durrani , and R. Pandey , “Porfimer Sodium Versus PS785 for Photodynamic Therapy (PDT) of Lung Cancer Xenografts in Mice,” Journal of Surgical Research 263 (2021): 245–250. 33713956 10.1016/j.jss.2020.12.067

[17] J. Usuda , H. Tsutsui , H. Honda , et al., “Photodynamic Therapy for Lung Cancers Based on Novel Photodynamic Diagnosis Using Talaporfin Sodium (NPe6) and Autofluorescence Bronchoscopy,” Lung Cancer 58 (2007): 317–323. 17698240 10.1016/j.lungcan.2007.06.026

[18] D. Bhattacharya , S. Datta , A. Das , K. C. Halder , and S. Chattopadhyay , “Primary Pulmonary Synovial Sarcoma: A Case Report and Review of Literature,” International Journal of Applied & Basic Medical Research 6 (2016): 63–65. 26958527 10.4103/2229-516X.174019 PMC4765279

[19] H. Zeren , C. A. Moran , S. Suster , N. F. Fishback , and M. N. Koss , “Primary Pulmonary Sarcomas With Features of Monophasic Synovial Sarcoma: A Clinicopathological, Immunohistochemical, and Ultrastructural Study of 25 Cases,” Human Pathology 26 (1995): 474–480. 7750931 10.1016/0046-8177(95)90242-2

[20] C. Liu , “Case of the Season: Primary Pulmonary Collision Tumor Consisting of Carcinoid and High‐Grade Spindle‐Cell Sarcoma,” Seminars in Roentgenology 46 (2011): 170–172. 21726700 10.1053/j.ro.2011.02.001

[21] H. J. Siegel , W. Sessions , M. A. Casillas , N. Said‐Al‐Naief , P. H. Lander , and R. Lopez‐Ben , “Synovial Sarcoma: Clinicopathologic Features, Treatment, and Prognosis,” Orthopedics 30 (2007): 1020–1025 quiz 1026‐7. 18198773 10.3928/01477447-20071201-15

[22] S. Stacchiotti and B. A. Van Tine , “Synovial Sarcoma: Current Concepts and Future Perspectives,” JCO 36 (2018): 180–187. 10.1200/JCO.2017.75.1941 29220290

[23] R. J. Lobb , K. S. Visan , L.‐Y. Wu , et al., “An Epithelial‐to‐Mesenchymal Transition Induced Extracellular Vesicle Prognostic Signature in Non‐Small Cell Lung Cancer,” Communications Biology 6 (2023): 68. 36653467 10.1038/s42003-022-04350-4 PMC9849257

[24] E. Baranov , M. J. McBride , A. M. Bellizzi , et al., “A Novel SS18‐SSX Fusion‐Specific Antibody for the Diagnosis of Synovial Sarcoma,” American Journal of Surgical Pathology 44 (2020): 922–933. 32141887 10.1097/PAS.0000000000001447 PMC7289668

[25] H. Y. Woo , “Biphasic Synovial Sarcoma With a Striking Morphological Divergence From the Main Mass to Lymph Node Metastasis,” Medicine (Baltimore) 101 (2022): e28481. 35029897 10.1097/MD.0000000000028481 PMC8735718

[26] S. S. Burks , R. C. Puffer , I. Cajigas , et al., “Synovial Sarcoma of the Nerve‐Clinical and Pathological Features: Case Series and Systematic Review,” Neurosurgery 85 (2019): E975–E991. 31435657 10.1093/neuros/nyz321 PMC6891799

[27] M. Miettinen , J. Limon , A. Niezabitowski , and J. Lasota , “Calretinin and Other Mesothelioma Markers in Synovial Sarcoma: Analysis of Antigenic Similarities and Differences With Malignant Mesothelioma,” American Journal of Surgical Pathology 25 (2001): 610–617. 11342772 10.1097/00000478-200105000-00007

[28] K. Nagao , H. Ito , and H. Yoshida , “Chromosomal Translocation t(X;18) in Human Synovial Sarcomas Analyzed by Fluorescence In Situ Hybridization Using Paraffin‐Embedded Tissue,” American Journal of Pathology 148 (1996): 601–609. 8579122 PMC1861692

[29] W. Wang , P. Hodkinson , F. McLaren , et al., “Histologic Assessment of Tumor‐Associated CD45+ Cell Numbers Is an Independent Predictor of Prognosis in Small Cell Lung Cancer,” Chest 143 (2013): 146–151. 22847040 10.1378/chest.12-0681

[30] S. Dennison , E. Weppler , and G. Giacoppe , “Primary Pulmonary Synovial Sarcoma: A Case Report and Review of Current Diagnostic and Therapeutic Standards,” Oncologist 9 (2004): 339–342. 15169989 10.1634/theoncologist.9-3-339

[31] G. Treglia , C. Caldarella , and S. Taralli , “A Rare Case of Primary Pulmonary Synovial Sarcoma in a Pediatric Patient Evaluated by (18)F‐FDG PET/CT,” Clinical Nuclear Medicine 39 (2014): e166–e168. 24536091 10.1097/RLU.0b013e318286bade

[32] S. P. Kuruva , S. Bala , M. L. Konatam , A. K. Karnam , L. S. Maddali , and S. Gundeti , “Clinicopathological Features, Treatment and Survival Outcomes of Synovial Sarcoma,” South Asian Journal of Cancer 07 (2018): 270–272. 10.4103/sajc.sajc_269_17 PMC6190396 30430100

[33] I. Yakavets , I. Yankovsky , T. Zorina , M. Belevtsev , L. Bezdetnaya , and V. Zorin , “Modulation of Temoporfin Distribution in Blood by Β‐Cyclodextrin Nanoshuttles,” Pharmaceutics 13 (2021): 1054. 34371745 10.3390/pharmaceutics13071054 PMC8308962

[34] Y.‐J. Hsieh , K.‐Y. Chien , I.‐F. Yang , et al., “Oxidation of Protein‐Bound Methionine in Photofrin‐Photodynamic Therapy‐Treated Human Tumor Cells Explored by Methionine‐Containing Peptide Enrichment and Quantitative Proteomics Approach,” Scientific Reports 7 (2017): 1370. 28465586 10.1038/s41598-017-01409-9 PMC5431048

[35] W. T. Lee , J. Yoon , S. S. Kim , et al., “Combined Antitumor Therapy Using In Situ Injectable Hydrogels Formulated with Albumin Nanoparticles Containing Indocyanine Green, Chlorin e6, and Perfluorocarbon in Hypoxic Tumors,” Pharmaceutics 14 (2022): 148. 35057044 10.3390/pharmaceutics14010148 PMC8781012

[36] D. E. J. G. J. Dolmans , D. Fukumura , and R. K. Jain , “Photodynamic Therapy for Cancer,” Nature Reviews. Cancer 3 (2003): 380–387. 12724736 10.1038/nrc1071

[37] H. Singh , B. S. Benn , C. Jani , M. Abdalla , and J. S. Kurman , “Photodynamic Therapy for Treatment of Recurrent Adenocarcinoma of the Lung with Tracheal Oligometastasis,” Respiratory Medicine Case Reports 37 (2022): 101620. 35330589 10.1016/j.rmcr.2022.101620 PMC8938912

[38] L. Xu , P. Wang , Y. Zhang , M. Wang , Y. Li , and W. Dang , “Study on Chronic Obstructive Pulmonary Disease and Lung Cancer: Web of Science‐Based Bibliometric and Visual Analysis,” International Journal of General Medicine 15 (2022): 7523. 36196373 10.2147/IJGM.S370781 PMC9527034

[39] J. LoCicero , M. Metzdorff , and C. Almgren , “Photodynamic Therapy in the Palliation of Late Stage Obstructing Non‐Small Cell Lung Cancer,” Chest 98 (1990): 97–100. 1694475 10.1378/chest.98.1.97

[40] K. Moghissi , K. Dixon , M. Stringer , T. Freeman , A. Thorpe , and S. Brown , “The Place of Bronchoscopic Photodynamic Therapy in Advanced Unresectable Lung Cancer: Experience of 100 Cases,” European Journal of Cardio‐Thoracic Surgery 15 (1999): 1–6. 10077365 10.1016/s1010-7940(98)00295-4

[41] H. Kato , C. Konaka , M. Saito , et al., “Laser Photodynamic Therapy With Hematoporphyrin Derivative in Lung Cancer,” Nihon Geka Gakkai Zasshi 86 (1985): 1059–1063. 4088208

[42] C. B. Simone , J. S. Friedberg , E. Glatstein , et al., “Photodynamic Therapy for the Treatment of Non‐small Cell Lung Cancer,” Journal of Thoracic Disease 4 (2012): 63–75. 22295169 10.3978/j.issn.2072-1439.2011.11.05 PMC3256541

[43] A. I. Arsen'ev , S. V. Kanaev , A. S. Barchuk , et al., “Use of Endotracheobronchial Surgery in Conjunction With Radiochemotherapy for Advanced Non‐Small Lung Cancer,” Voprosy Onkologii 53 (2007): 461–467. 17969412

[44] U. Wollina , A. Bitel , A. Vojvodic , and T. Lotti , “Rosacea Flare‐Up after Photodynamic Therapy (PDT) for Field Cancerization and a Review on Adverse Events With PDT in General,” Open Access Macedonian Journal of Medical Sciences 7 (2019): 2998–3001. 31850108 10.3889/oamjms.2019.536 PMC6910807

[45] D. Kessel , W. J. Cho , J. Rakowski , H. E. Kim , and H.‐R. C. Kim , “Characteristics of an Impaired PDT Response,” Photochemistry and Photobiology 97 (2021): 837–840. 33570777 10.1111/php.13397 PMC8277670

[46] T. Okunaka , T. Hiyoshi , K. Furukawa , et al., “Lung Cancers Treated With Photodynamic Therapy and Surgery,” Diagnostic and Therapeutic Endoscopy 5 (1999): 155–160. 18493497 10.1155/DTE.5.155 PMC2362632

[47] J. S. Friedberg , R. Mick , J. P. Stevenson , et al., “Phase II Trial of Pleural Photodynamic Therapy and Surgery for Patients With Non‐Small‐Cell Lung Cancer With Pleural Spread,” Journal of Clinical Oncology 22 (2004): 2192–2201. 15169808 10.1200/JCO.2004.07.097

[48] P. Ross , J. Grecula , T. Bekaii‐Saab , M. Villalona‐Calero , G. Otterson , and C. Magro , “Incorporation of Photodynamic Therapy as an Induction Modality in Non‐Small Cell Lung Cancer,” Lasers in Surgery and Medicine 38 (2006): 881–889. 17115382 10.1002/lsm.20444

[49] X.‐X. Liu , G.‐H. Lin , and B.‐C. Wang , “A Bayesian Network Analysis of Immunotherapy and Taxane Chemotherapy as Second‐ or Later‐Line Treatments in Non‐Small Cell Lung Cancer,” Medicine (Baltimore) 101 (2022): e31751. 36397323 10.1097/MD.0000000000031751 PMC9666094

[50] F. Jin , H. Wang , Q. Li , et al., “Clinical Application of Photodynamic Therapy for Malignant Airway Tumors in China,” Thoracic Cancer 11 (2020): 181–190. 31760687 10.1111/1759-7714.13223 PMC6938770

[51] C. B. Simone and K. A. Cengel , “Photodynamic Therapy for Lung Cancer and Malignant Pleural Mesothelioma,” Seminars in Oncology 41 (2014): 820–830. 25499640 10.1053/j.seminoncol.2014.09.017 PMC4272687

[52] Z. Shen , Q. Ma , X. Zhou , et al., “Strategies to Improve Photodynamic Therapy Efficacy by Relieving the Tumor Hypoxia Environment,” NPG Asia Materials 13 (2021): 39.

[53] S. Lam , E. C. Kostashuk , E. P. Coy , et al., “A Randomized Comparative Study of the Safety and Efficacy of Photodynamic Therapy Using Photofrin II Combined with Palliative Radiotherapy versus Palliative Radiotherapy Alone in Patients with Inoperable Obstructive Non‐Small Cell Bronchogenic Carcinoma,” Photochemistry and Photobiology 46 (1987): 893–897. 2450381 10.1111/j.1751-1097.1987.tb04865.x

[54] X. W. Chen and X. L. Song , “Application and Progress of Photodynamic Therapy in Combination Therapy of Lung Cancer,” Zhonghua jie he hu xi za zhi= Zhonghua jiehe he huxi zazhi= Chinese journal of tuberculosis and respiratory diseases 46 (2023): 424–429. 10.3760/cma.j.cn112147-20221214-00974 36990709

